# Genome-wide and expression analysis of protein phosphatase 2C in rice and Arabidopsis

**DOI:** 10.1186/1471-2164-9-550

**Published:** 2008-11-20

**Authors:** Tongtong Xue, Dong Wang, Shizhong Zhang, Juergen Ehlting, Fei Ni, Stephen Jakab, Chengchao Zheng, Yuan Zhong

**Affiliations:** 1 State Key Laboratory of Crop Biology, College of Life Sciences, Shandong Agricultural University, Taian, Shandong 271018, PR China; 2 Biology Department, Millersville University of Pennsylvania, 288 Roddy Hall, 50 E Frederick St, PO Box 1002, Millersville PA, 17551-0302, USA; 3 Department of Chemistry, Fudan University, 220 Handan Road, Shanghai 200433, PR China; 4 Institutes of Biomedical Sciences, Fudan University, 130 Dongan Road, Shanghai 200032, PR China; 5 Centre for Forest Biology & Department of Biology, University of Victoria, Victoria BC, V8W 3N5, Canada

## Abstract

**Background:**

The protein phosphatase 2Cs (PP2Cs) from various organisms have been implicated to act as negative modulators of protein kinase pathways involved in diverse environmental stress responses and developmental processes. A genome-wide overview of the PP2C gene family in plants is not yet available.

**Results:**

A comprehensive computational analysis identified 80 and 78 PP2C genes in *Arabidopsis thaliana *(AtPP2Cs) and *Oryza sativa *(OsPP2Cs), respectively, which denotes the PP2C gene family as one of the largest families identified in plants. Phylogenic analysis divided PP2Cs in Arabidopsis and rice into 13 and 11 subfamilies, respectively, which are supported by the analyses of gene structures and protein motifs. Comparative analysis between the PP2C genes in Arabidopsis and rice identified common and lineage-specific subfamilies and potential 'gene birth-and-death' events. Gene duplication analysis reveals that whole genome and chromosomal segment duplications mainly contributed to the expansion of both OsPP2Cs and AtPP2Cs, but tandem or local duplication occurred less frequently in Arabidopsis than rice. Some protein motifs are widespread among the PP2C proteins, whereas some other motifs are specific to only one or two subfamilies. Expression pattern analysis suggests that 1) most PP2C genes play functional roles in multiple tissues in both species, 2) the induced expression of most genes in subfamily A by diverse stimuli indicates their primary role in stress tolerance, especially ABA response, and 3) the expression pattern of subfamily D members suggests that they may constitute positive regulators in ABA-mediated signaling pathways. The analyses of putative upstream regulatory elements by two approaches further support the functions of subfamily A in ABA signaling, and provide insights into the shared and different transcriptional regulation machineries in dicots and monocots.

**Conclusion:**

This comparative genome-wide overview of the PP2C family in Arabidopsis and rice provides insights into the functions and regulatory mechanisms, as well as the evolution and divergence of the PP2C genes in dicots and monocots. Bioinformatics analyses suggest that plant PP2C proteins from different subfamilies participate in distinct signaling pathways. Our results have established a solid foundation for future studies on the functional divergence in different PP2C subfamilies.

## Background

The reversible phosphorylation of proteins is a fundamental mechanism by which living organisms modulate cellular processes including cell cycle events, growth factor responses, hormone and environmental stimuli responses, metabolic control, and developmental processes [1-5]. Protein phosphatases (PPs), by reversing the action of protein kinases, provide modulations of protein phosphoregulation. PPs can be grouped into two major classes based on substrate specificity: protein tyrosine phosphatases and protein serine/threonine phosphatases [6]. Based on enzymological criteria using specific inhibitors and activators, most serine/threonine protein phosphatases have been placed into two subcategories: the protein phosphatase 1 (PP1) and protein phosphatase 2 (PP2) [6]. PP2 proteins were further distinguished by metal ion requirements: while protein phosphatases 2A (PP2As) have no ion requirement, PP2Bs require Ca^2+ ^and PP2Cs require Mg^2+ ^or Mn^2+^. Alternatively, based on both distinct amino acid sequences and crystal structures, protein serine/threonine phosphatases can be classified into the Phosphor-protein phosphatase (PPP) and the Mg^2+^- or Mn^2+^- dependent protein phosphatase (PPM) families. The PPP family covers the PP1, PP2A and PP2B groups, whereas the PPM family includes the PP2C group and also pyruvate dehydrogenase phosphatases [7].

Members of the PP2C group from various organisms have been implicated in acting as negative modulators of protein kinase pathways activated by diverse environmental stresses or developmental signaling cascades [8-15]. In plants, several PP2Cs have been described as negative regulators within the abscisic acid (ABA) mediated signaling network [16-20]. Two dominant ABA insensitive mutants, *abi1-1 *and *abi2-1*, are impaired in PP2C genes (*ABI1 *and *ABI2*). Proteins encoded by these genes are important for plant tolerance to several abiotic stresses including salt, drought, and freezing [16,21-30]. Also *HAB1 *and *PP2CA*, homologous PP2Cs from Arabidopsis, are negative regulators involved in ABA signaling [19,20,23,31-33]. Anti-sense mediated down-regulation of *PP2CA *genes accelerates plant development and leads to freezing tolerance [17]. Two PP2C genes (*FsPP2C1 *and *FsPP2C2*) were isolated from beech seeds (*Fagus sylvatica*). Ectopic expression of *FsPP2C1 *in Arabidopsis, which shows high sequence similarity with *ABI1 *and *ABI2*, confers ABA insensitivity during seed dormancy and germination under unfavorable conditions, indicating that FsPP2C1 also acts as a negative regulator of ABA signaling [18]. However, ectopic expression of *FsPP2C2 *in Arabidopsis resulted in enhanced sensitivity to ABA and abiotic stress in seeds and vegetative tissues and caused a dwarf phenotype and delayed flowering, which suggests that FsPP2C2 is a positive regulator of ABA [34].

Other plant PP2C members, such as MP2C from *Mesembryanthemum crystallinum*, act as counterparts of the mitogen-activated protein kinase pathway during salt stress or wounding [35]. A kinase-associated protein phosphatase 2C (KAPP) was originally identified as an interacting protein of the receptor-like kinase CLAVATA1 (CLV1). KAPP acts as a negative modulator of CLV1 and is required for the proper balance between cell proliferation and differentiation in Arabidopsis shoots and floral meristems [36,37]. KAPP is also a component of a novel Na^+ ^acclimation pathway [38]. Moreover, *POLTERGEIST *(*POL*), encoding another PP2C in Arabidopsis, was also identified as a modulator of the CLAVATA pathways controlling stem cell identity in meristems [39].

The availability of the complete *Arabidopsis *and *Oryza sativa *genome sequences allows a timely, systematic genome-wide comparative analysis of gene families between monocots and eudicots [40-44]. Previously, 76 genes from Arabidopsis were identified as PP2C candidates [4]; however, important features like gene structures, protein motifs and gene duplication events were not explored, which is critical to understanding the characteristics of the Arabidopsis PP2C gene (*AtPP2C*) family. Moreover, the rice PP2C gene (*OsPP2C*) family has not been analyzed in detail, and the phylogenetic relationship of the PP2C genes between Arabidopsis and rice remains poorly understood.

In this study, we first identified all the PP2C genes from Arabidopsis and rice, and then conducted phylogenetic analyses to divide them into subfamilies. We further compared the rice and Arabidopsis PP2C genes to identify both shared and specific subfamilies, as well as potential gene birth-and-death events. Following the analysis of gene structure and protein motifs, we traced gene duplication events that likely contributed to the expansion of the PP2C family. The expression patterns of the PP2Cs from rice and Arabidopsis were analyzed and compared, in order to shed light on their functional roles, especially in ABA-signaling and stress tolerance. The upstream regulatory regions of the subfamily A members were finally analyzed in order to provide insight into the transcriptional regulation machineries that define the temporal and spatial expression and the stimuli responses.

## Results and discussion

### Identification of PP2C genes in Arabidopsis and rice

To uncover the entire family of genes coding for AtPP2C proteins in Arabidopsis, we performed multiple keyword searches to obtain PP2C protein sequences from several databases. Sequences from these hits were used as queries employing BLAST algorithms to search against the corresponding Arabidopsis sequence data sets (see Methods for details). A custom Perl program was used to parse the results and to exclude the redundant entries from the initial data set. Subsequently, the SMART [45] and Pfam [46] motif identification tools were used to identify predicted PP2C domains in all these candidate proteins. As a result of this filtering, 109 non-redundant putative proteins with PP2C domains encoded by 80 genes were extracted from the Arabidopsis genome annotation (Table 1).

**Table 1:** The PP2C gene family of Arabidopsis

Gene identifier	Gene name	Size (aa)	Mass (kDa)	IP	Other name	Alternative splicing	Expression*
AT1G03590	AtPP2C1	447	49368	5.0807			A B C
AT1G07160	AtPP2C2	380	40709	7.3753			A B D
AT1G07430	AtPP2C3	442	49763	6.1573			A B C D
AT1G07630	AtPP2C4	662	74275	5.0448	PLL5		A B C D
AT1G09160	AtPP2C5	428	45781	6.6222		2	A B C D
AT1G16220	AtPP2C6	491	54354	5.2075			A B
AT1G17550	AtPP2C7	511	56062	4.4838	HAB2		A B C D
AT1G18030	AtPP2C8	351	38486	7.3468		2	A C D
AT1G22280	AtPP2C9	281	30722	7.1987		2	A B C D
AT1G34750	AtPP2C10	282	30984	7.6896			A B C D
AT1G43900	AtPP2C11	371	40583	5.4926			A B C D
AT1G47380	AtPP2C12	428	46422	5.0749			A B C D
AT1G48040	AtPP2C13	383	42106	4.5823			A B C D
AT1G67820	AtPP2C14	445	49353	8.3146			A B C D
AT1G68410	AtPP2C15	436	47041	8.357		2	A B C D
AT1G72770	AtPP2C16	511	55745	4.473	HAB1	2	A B C D
AT1G78200	AtPP2C17	283	30854	9.0702		2	A B C D
AT1G79630	AtPP2C18	504	55604	4.5136		3	A B C D
AT2G20050	AtPP2C19	514	56686	5.0617			A C
AT2G20630	AtPP2C20	290	31847	6.2456	AtPPC3	2	A B C D
AT2G25070	AtPP2C21	355	39354	4.5923			A B C D
AT2G25620	AtPP2C22	392	42674	5.3137			A B C D
AT2G28890	AtPP2C23	654	72483	4.9016	PLL4		A B C D
AT2G29380	AtPP2C24	362	40297	5.2089			A D
AT2G30020	AtPP2C25	396	42420	7.9156			B C D
AT2G30170	AtPP2C26	298	32285	4.9442		2	A B C D
AT2G33700	AtPP2C27	380	41758	5.0757			A B C D
AT2G34740	AtPP2C28	239	26362	5.9641			A B C
AT2G35350	AtPP2C29	783	86523	4.9655	PLL1		B C
AT2G40180	AtPP2C30	390	42596	6.53	ATHPP2C5		A B C D
AT2G40860	AtPP2C31	658	72854	5.904			A B C D
AT2G46920	AtPP2C32	856	95572	5.0992	POL	2	A B C D
AT3G02750	AtPP2C33	527	57675	4.6678		3	A B C D
AT3G05640	AtPP2C34	358	39780	6.9143		2	A B C D
AT3G06270	AtPP2C35	348	38330	4.4284			B C D
AT3G09400	AtPP2C36	650	72381	6.6816	PLL3		B
AT3G11410	AtPP2C37	399	43350	6.314	ATPP2CA		A B C D
AT3G12620	AtPP2C38	385	42852	9.8536		2	A B C D
AT3G15260	AtPP2C39	289	31630	5.6062		2	A B C D
AT3G16560	AtPP2C40	493	53614	4.7551			A B C D
AT3G16800	AtPP2C41	351	38601	7.8321		3	A B C D
AT3G17090	AtPP2C42	384	42490	6.9292		2	B C D
AT3G17250	AtPP2C43	422	47599	4.8404			A B C D
AT3G23360	AtPP2C44	256	29168	7.546			B
AT3G27140	AtPP2C45	245	26567	8.2286			
AT3G51370	AtPP2C46	379	42180	8.5835		2	A B C D
AT3G51470	AtPP2C47	361	39486	5.4774			A B D
AT3G55050	AtPP2C48	384	42851	7.2137		2	A B C D
AT3G62260	AtPP2C49	384	41969	4.2802		2	A B C D
AT3G63320	AtPP2C50	423	46803	6.1909			A
AT3G63340	AtPP2C51	528	58355	7.8728			B C D
AT4G03415	AtPP2C52	453	50258	6.1037			B C D
AT4G08260	AtPP2C53	212	23323	6.5128			A
AT4G11040	AtPP2C54	295	31788	6.7789			D
AT4G16580	AtPP2C55	467	50297	8.2099			B C D
AT4G26080	AtPP2C56	434	47506	5.9831	ABI1		A B C D
AT4G27800	AtPP2C57	388	42720	6.0154	PPH1	3	A B C D
AT4G28400	AtPP2C58	283	31019	8.1645			A B C D
AT4G31750	AtPP2C59	311	33248	4.4332			A B C D
AT4G31860	AtPP2C60	357	39203	4.7506		2	A B C D
AT4G32950	AtPP2C61	326	326	6.5793			A B
AT4G33500	AtPP2C62	724	78924	4.375			A B C D
AT4G33920	AtPP2C63	380	42319	9.5857			A B C D
AT4G38520	AtPP2C64	400	44124	7.9157		2	A B C D
AT5G01700	AtPP2C65	382	42005	5.4977		2	A B C D
AT5G02400	AtPP2C66	674	74839	6.0726	PLL2		B
AT5G02760	AtPP2C67	370	41335	9.1472			A B C D
AT5G06750	AtPP2C68	393	44141	6.8836			A B C D
AT5G10740	AtPP2C69	354	38037	5.1697			A B C D
AT5G19280	AtPP2C70	581	64912	6.8997	KAPP,RAG1		A B C D
AT5G24940	AtPP2C71	447	48120	5.657			A B C D
AT5G26010	AtPP2C72	331	36522	5.1209			A B C
AT5G27930	AtPP2C73	373	41584	7.3002	AtPPC6	2	A B C D
AT5G36250	AtPP2C74	448	49314	7.5011			A B C D
AT5G51760	AtPP2C75	416	46043	5.2877			A C
AT5G53140	AtPP2C76	420	45787	4.7268			A B C D
AT5G57050	AtPP2C77	423	46307	6.2578	ABI2		A B C D
AT5G59220	AtPP2C78	413	45538	6.4064			A B C D
AT5G66080	AtPP2C79	385	42963	8.07			A B C D
AT5G66720	AtPP2C80	414	43874	7.7736		2	B C D

*Evidence for gene expression inferred from (A) community microarray data, (B) massively parallel signature sequencing (MPSS), (C) EST clones, and/or (D) full-length cDNA clones.

To explore the occurrence and size of the PP2C gene family in rice, all predicted AtPP2C protein sequences were initially used as query sequences to search against the TIGR rice database [47] using BLASTP. Multiple BLAST searches were then performed in several rice databases using rice protein sequences with significant hits as queries (see Methods for details). After confirming the presence of PP2C catalytic domains using SMART and Pfam, 111 putative proteins encoded by 78 genes were identified as PP2C members in rice (Table 2). While scanning for protein catalytic domains, additional specific motifs were found in the regions outside of the PP2C catalytic domain in both Arabidopsis and rice PP2Cs. Transmembrane spanning regions were found in AT1G43900, Os01g37130 and Os02g05630, protein kinase catalytic domains were present in AT2G40860, Os01g36080 and Os11g37540, and a forkhead-associated domain (FHA) was identified in At5G19280 (KAPP).

**Table 2:** The PP2C gene family of rice

Gene	Gene name	Size(aa)	Mass(kDa)	pI	alternative splicing	Expression*
LOC_Os01g07090	OsPP2C01	332	35.43	4.69		B C D E
LOC_Os01g19130	OsPP2C02	381	41.82	4.59		B C D E
LOC_Os01g32964	OsPP2C03	335	35.90	7.91		B C D E
LOC_Os01g36080	OsPP2C04	658	73.19	6.26	4	B C D E
LOC_Os01g37130	OsPP2C05	390	41.74	5.51	2	B C D E
LOC_Os01g40094	OsPP2C06	468	48.63	4.77		B C
LOC_Os01g43100	OsPP2C07	392	41.81	8.41		B C
LOC_Os01g46760	OsPP2C08	404	43.00	5.44		B C D
LOC_Os01g62760	OsPP2C09	415	43.99	5.25		B C D E
LOC_Os02g05630	OsPP2C10	349	37.57	5.02	3	B C D E
LOC_Os02g08364	OsPP2C11	363	39.71	5.04		B C D E
LOC_Os02g13100	OsPP2C12	390	41.00	5.88		B C D E
LOC_Os02g15594	OsPP2C13	364	38.96	5.53		C D
LOC_Os02g27220	OsPP2C14	519	55.29	8.71		B C D E
LOC_Os02g35910	OsPP2C15	443	48.01	5.72		B C D
LOC_Os02g38580	OsPP2C16	522	56.26	4.66		C D
LOC_Os02g38690	OsPP2C17	764	82.81	4.81		B
LOC_Os02g38710	OsPP2C18	805	84.98	4.21		B C D
LOC_Os02g38780	OsPP2C19	653	71.41	4.65	3	B C D
LOC_Os02g38804	OsPP2C20	518	56.55	4.51		B C
LOC_Os02g39410	OsPP2C21	341	53.76	10.82	3	B C D
LOC_Os02g39480	OsPP2C22	582	63.30	4.88		
LOC_Os02g42250	OsPP2C23	320	34.73	4.99		
LOC_Os02g42270	OsPP2C24	316	33.45	5.28		
LOC_Os02g46080	OsPP2C25	388	43.01	8.55	2	B D
LOC_Os02g46490	OsPP2C26	597	64.83	5.75		B C D E
LOC_Os02g55560	OsPP2C27	185	39.27	4.9	2	B C D E
LOC_Os03g04430	OsPP2C28	400	44.29	8.77	3	B C D
LOC_Os03g10950	OsPP2C29	393	42.51	8.22		B C D
LOC_Os03g16170	OsPP2C30	405	43.28	5.71		B C D E
LOC_Os03g16760	OsPP2C31	632	68.74	6.39		B C D
LOC_Os03g18150	OsPP2C32	392	41.31	6.37		B C E
LOC_Os03g18970	OsPP2C33	433	45.83	6.69		B C D
LOC_Os03g55320	OsPP2C34	381	41.75	9.79	3	B C D E
LOC_Os03g60650	OsPP2C35	640	69.18	5.31		B C D E
LOC_Os03g61690	OsPP2C36	387	42.02	7.25	2	B C D
LOC_Os04g08560	OsPP2C37	435	44.81	7.24		B C
LOC_Os04g25570	OsPP2C38	461	49.69	6.78		B C D
LOC_Os04g33080	OsPP2C39	521	57.32	4.57		B C E
LOC_Os04g37660	OsPP2C40	462	48.71	6.32	2	B C
LOC_Os04g37904	OsPP2C41	285	31.01	6.25	3	B C E
LOC_Os04g42260	OsPP2C42	353	38.17	6.83		B C
LOC_Os04g49490	OsPP2C43	389	43.19	6.51		B C
LOC_Os04g52000	OsPP2C44	322	34.77	8.66		B C D E
LOC_Os04g56450	OsPP2C45	283	30.58	4.75	2	B C D E
LOC_Os05g02110	OsPP2C46	594	63.48	5.28		B
LOC_Os05g04360	OsPP2C47	390	42.38	4.55		B C D E
LOC_Os05g29030	OsPP2C48	354	38.10	7.38	2	B C D
LOC_Os05g38290	OsPP2C49	417	43.78	8.33		B C E
LOC_Os05g46040	OsPP2C50	388	41.55	5.97	2	B C
LOC_Os05g49730	OsPP2C51	382	40.35	7.83		B C D
LOC_Os05g50970	OsPP2C52	492	52.92	4.49		B C D
LOC_Os05g51510	OsPP2C53	446	46.68	4.67	2	B C D E
LOC_Os06g08140	OsPP2C54	361	39.35	4.98		B C D
LOC_Os06g33530	OsPP2C55	354	38.29	10.56		B E
LOC_Os06g33549	OsPP2C56	353	37.81	8.48		B C E
LOC_Os06g39600	OsPP2C57	368	39.44	5.17	2	B C D
LOC_Os06g44210	OsPP2C58	369	40.59	4.71	2	B C D E
LOC_Os06g48300	OsPP2C59	328	34.94	4.39		B C D E
LOC_Os06g50380	OsPP2C60	393	43.58	8.65	2	B C D E
LOC_Os07g02330	OsPP2C61	378	40.62	8.24		B C D E
LOC_Os07g32380	OsPP2C62	291	31.74	6.97		B C
LOC_Os07g33230	OsPP2C63	224	24.68	7.05		B
LOC_Os07g37890	OsPP2C64	428	46.30	5.55		B C D E
LOC_Os07g45170	OsPP2C65	446	47.22	5.62		B C
LOC_Os08g39100	OsPP2C66	532	57.67	5		B C D
LOC_Os09g14540	OsPP2C67	368	39.39	6.83		B C D
LOC_Os09g15670	OsPP2C68	359	37.70	6.5		B C D
LOC_Os09g28560	OsPP2C69	423	45.81	5.34		C
LOC_Os09g38550	OsPP2C70	352	38.47	5.08	4	B C D E
LOC_Os10g22460	OsPP2C71	466	48.49	4.08		B C D
LOC_Os10g39780	OsPP2C72	335	36.76	6.84	4	B C D E
LOC_Os11g01790	OsPP2C73	421	46.26	5.85		B C D
LOC_Os11g13820	OsPP2C74	398	41.35	6.64		C
LOC_Os11g22404	OsPP2C75	433	47.11	6.12		B C D
LOC_Os11g37540	OsPP2C76	1116	127.19	6.98		B C E
LOC_Os12g09640	OsPP2C77	422	435.57	7.32		B C
LOC_Os12g39120	OsPP2C78	393	43.53	8.61		B C D

*Evidence for gene expression inferred from (B) massively parallel signature sequencing (MPSS), (C) EST clones, (D) full-length cDNA clones and (E) serial analysis of gene expression (SAGE).

Previously, 76 genes have been identified as AtPP2C candidate genes [4], all of which were present in our gene set except for AT1G75010, which does not contain a PP2C catalytic domain according to SMART and Pfam, and was thus excluded from our analysis. Twenty-five and twenty one alternative splicing variants were found in the Arabidopsis and rice PP2C gene families, respectively (Table 1 and 2). Because alternative splice variants share a common coding sequence, they are closely related to each other in multiple sequence alignment and phylogenetic analysis. Thus we selected only a single variant for further analysis. Despite the differences in genome sizes between Arabidopsis and rice (125 Mb and 389 Mb, respectively), the two plant species appear to have a very similar number of genes encoding PP2C proteins. Compared to other gene families analyzed in rice and Arabidopsis, the PP2C gene family is one of the largest families in the plant kingdom. Only six PP2Cs were found in the yeast (*Saccharomyces cerevisiae*) genome, and no more than fifteen are present in the human genome [48,49]. The high number of PP2C genes present in the Arabidopsis and rice genome highlights the importance of the PP2C class of protein phosphatases in plants, and suggests that plant PP2Cs may have narrower substrate range than other organisms [16,34].

### Phylogenetic and comparative analysis of PP2C family in Arabidopsis and rice

**Figure 1:**
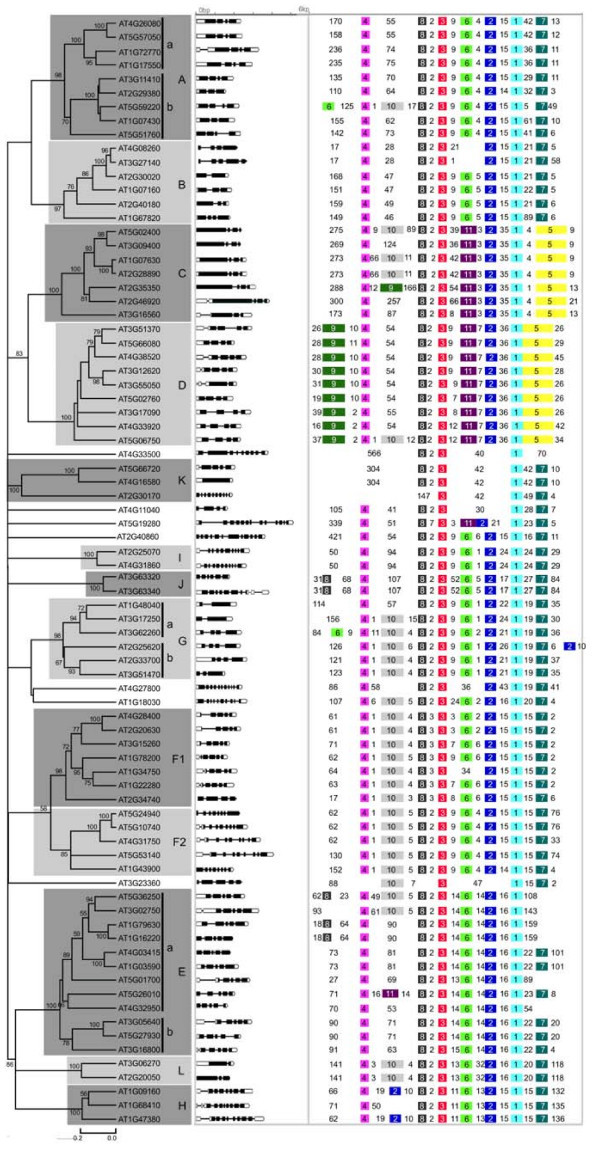
**An analytical view on the PP2C gene family in Arabidopsis**. The following parts are shown from left to right. Protein distance tree generated by neighbor-joining: The unrooted tree, constructed using Mega (4.0), summarizes the phylogenetic relationship among the 80 members of the PP2C family in Arabidopsis. The neighbor-joining tree was constructed using aligned full-length amino acid sequences. The tree shows the 13 major phylogenetic subfamilies (numbered A to L, and marked with different alternating tones of a gray background to make subfamily identification easier). The numbers beside the branches represent bootstrap values (≥ 50%) based on 1000 replicates that were used to group the 13 subfamilies. Gene structure: The gene structure is represented by white boxes symbolizing untranslated regions, black boxes symbolizing exons and spaces between boxes corresponding to introns. The sizes of exons, introns and untranslated regions are drawn to scale as indicated on top. Protein structure: Each colored box with a number represents the occurrence of a specific motif in the protein identified using the MEME motif search tool (the sequence and length of each motif is shown in Table 3). The order of motifs corresponds to their position in individual protein sequences. Numbers between colored boxes represent the amount of amino acid residues between the motifs in AtPP2C proteins.

Using the 80 PP2C full length amino acid sequences from Arabidopsis, a phylogenetic analysis was performed to construct a phylogenetic tree (Figure 1). The results showed that all but 7 AtPP2Cs can be divided into 13 subfamilies. Previously, 76 genes have been divided into 10 subfamilies (A-J) [4,50]. To be consistent with this past study, we adopted the same name for the nine common subfamilies (A, B, C, D, E, G, H, I, J). Since the members of subfamily F are separated into two subfamilies in our study, we named those two subfamilies F1 and F2. High bootstrap support is observed for each subfamily clade (85% for F2 and > 90% for the remainders), suggesting that the genes in the same subfamily share the same evolutionary origin. Three of the five novel members identified in this study (AT2G30170, AT4G16580, AT5G66720) form a new subfamily, which is designated K. The fourth (AT4G03415) is placed into subfamily E, and the last (AT4G33500) forms a single branch. Subfamily A, G and E are further divided into subgroups with at least 50% bootstrap support. Moreover, subfamilies C and D as well as subfamilies E, L and H constitute sister clades in a monophyletic cluster with high bootstrap support (83% and 86%, respectively), suggesting close evolutionary relationships between the respective subfamilies. Fifty of the AtPP2Cs form 25 paralogous sister pairs, 18 of which have very strong bootstrap support (> 90%).

**Figure 2:**
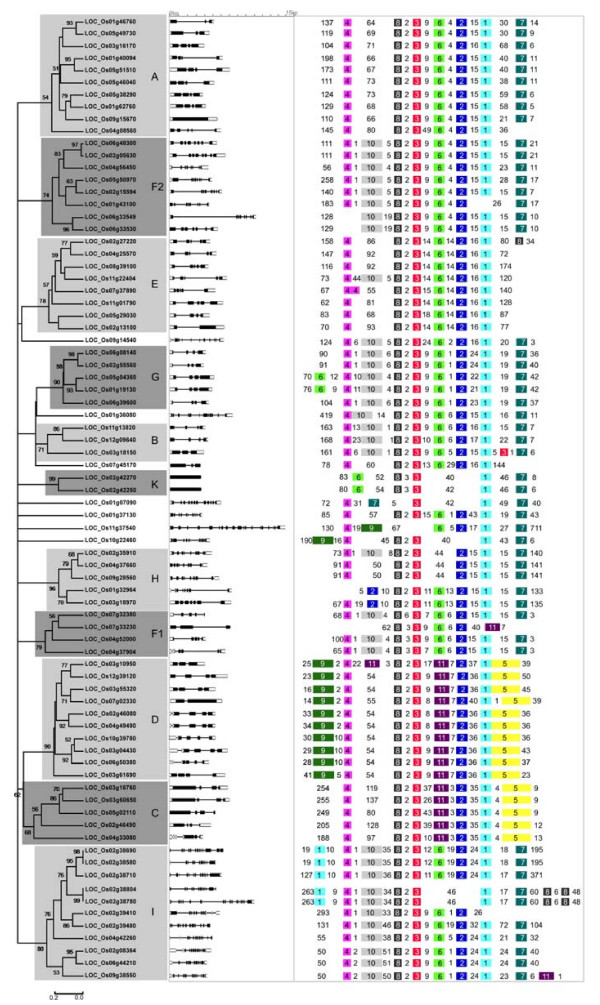
**An analytical view on the PP2C gene family in rice**. The following parts are shown from left to right. Protein distance tree: The unrooted tree was constructed using aligned full-length amino acid sequences and summarizes the phylogenetic relationship among the 78 members of PP2C family in rice. The tree shows the 11 major phylogenetic subfamilies (marked with different alternating tones of a gray background) with high bootstrap support value (≥ 50%) based on 1000 replicates. The same designations for gene families as in Figure 1 are used. Singleton rice sequences corresponding to the J and L clades from Arabidopsis are indicated. Gene structure: The gene structures are represented by white boxes symbolizing untranslated regions, black boxes symbolizing exons, and spaces between boxes corresponding to introns. Gene models are drawn to scale as indicated on top. Protein structure: Each colored box with a number represents a specific motif in the protein identified using the MEME motif search tool. The order of the motifs corresponds to their position within individual protein sequences. Numbers between the colored boxes indicate the number of amino acid residues between the motifs.

In order to evaluate the evolutionary relationship among the 78 rice PP2C proteins, we also performed a phylogenetic analysis based on their full length amino acid sequences (Figure 2). Eleven subfamilies are identified as monophyletic clades with at least 50% bootstrap support, and additional seven genes form single branches (Figure 2). Twenty-five sister pairs of paralogous OsPP2Cs are found, eleven of which had very strong bootstrap support (> 90%). Out results suggest a clear paralogous pattern of PP2C gene divergence by gene duplication both in monocots and eudicots.

Structural examination reveals that untranslated regions (UTRs) are present in 71 AtPP2C genes and 70 OsPP2C genes (Figure 1 and 2). The number of introns ranged from zero to twelve in AtPP2Cs and from zero to eighteen in OsPP2Cs. Most members within the same subfamily, both in Arabidopsis and rice, share a similar intron/exon structure and gene length. This conserved intron/exon structure in each subfamily supports their close evolutionary relationship and the introduced classification of subfamilies.

**Figure 3:**
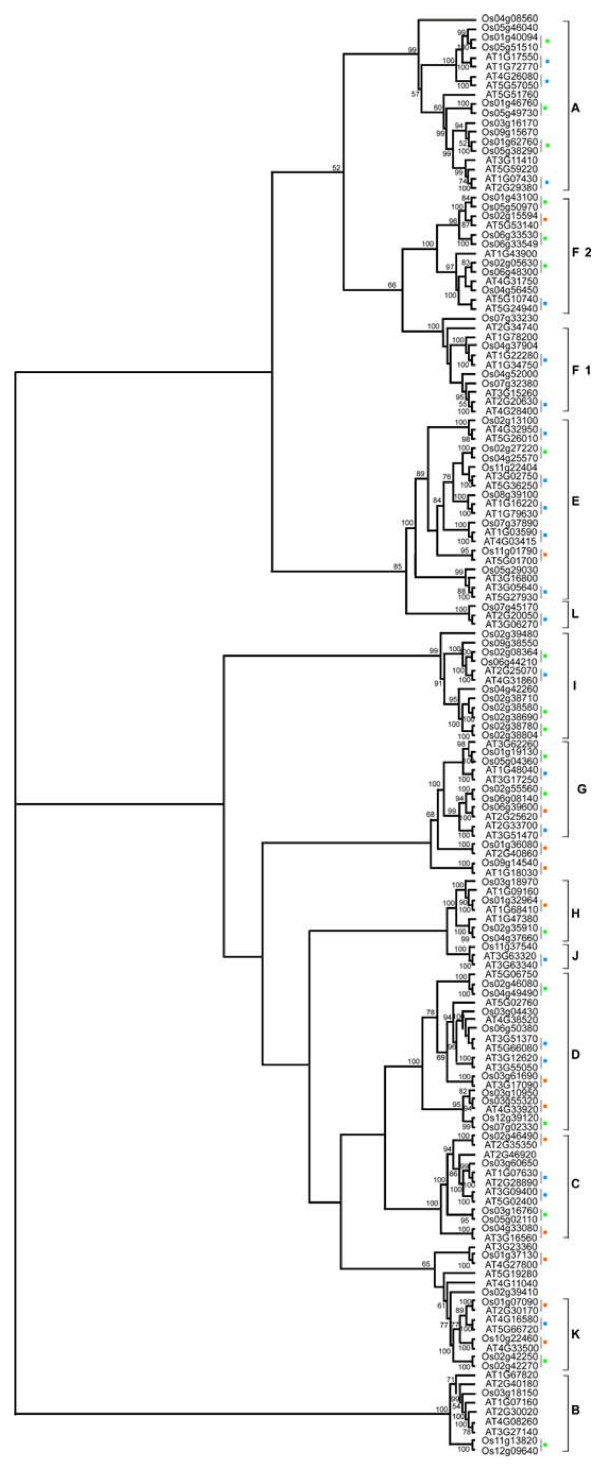
**Phylogenetic relationships of Arabidopsis and rice PP2C proteins**. The unrooted distance tree was inferred using the neighbor-joining method and is based on an alignment of the full-length amino acid sequences of the 158 Arabidopsis and rice genes listed in Table 1 and Table 2. The 13 phylogenetic subfamilies with high bootstrap support values are consistent with the neighbor-joining tree of Arabidopsis PP2C gene family (Figure 1). The numbers beside the branches indicate bootstrap values (≥ 50%) based on 1000 replicates that support the adjacent node. Putative otthologous pairs from Arabidopsis and rice are marked with orange squares. Paralogous gene pairs from Arabidopsis and rice are marked with blue and green squares, respectively.

We further investigated the relationship between the Arabidopsis and rice PP2C protein families by generating an alignment of the full-length protein sequences of 78 OsPP2Cs and 80 AtPP2Cs followed by the construction of a phylogenetic tree (Figure 3). While bootstrap values for internal nodes are low, the outer nodes allow the clustering of the PP2C genes of Arabidopsis and rice into 13 subfamilies with higher support values (> 98%). The tree topology, as well as the subfamily organization, resembles the individual trees from rice and Arabidopsis (Figure 1 and 2). The clades defining all 13 subfamilies contain members from both rice and Arabidopsis. Subfamilies J and L each contain only a single rice member, and are thus defined as singletons instead of families in rice. The remaining 11 rice subfamilies are named according to the PP2C subfamily nomenclature in Arabidopsis (Figure 2). Consistent with the AtPP2C gene family analysis, pretty high bootstrap support was also found for a clade combining subfamilies C and D in rice (62%), confirming the close evolutionary relationship between these two subfamilies in both eudicots and monocots. The rice and Arabidopsis genes in each subfamily are thus more closely related to each other than to the PP2C genes of the same species from a different subfamily, suggesting that an ancestral set of PP2C genes defining each subfamily already existed before the monocot-eudicot divergence. Twenty-six putative Arabidopsis/rice orthologs are identified in the tree with high statistical support (indicated by the orange squares in Figure 3). Furthermore, the phylogenetic construction of the PP2C family fits well with the model of birth-and-death evolution in the flowering plant lineage [51,52]. The branches with more than one AtPP2C or OsPP2C gene likely experienced gene birth due to gene duplication events, whereas the branches with only Arabidopsis or only rice PP2C members likely had gene death. Similarly, other large gene families also experienced birth-and-death evolution, such as the MADS-box gene family and the basic/helix-loop-helix (bHLH) transcription factor family analyzed in both rice and Arabidopsis [53,54].

### Evolution and divergence of PP2C genes in Arabidopsis and rice

Large segmental duplications of chromosomal regions during evolution, followed by gene loss, small-scale duplications and local rearrangements, have created the present complexities of plant genome [55]. Indications of several whole genome duplication have been found in the Arabidopsis genome [56-58]. Likewise, two rounds of large scale genome duplications have occurred in the lineage leading to rice, which cover most rice chromosomes [40,59].

**Figure 4:**
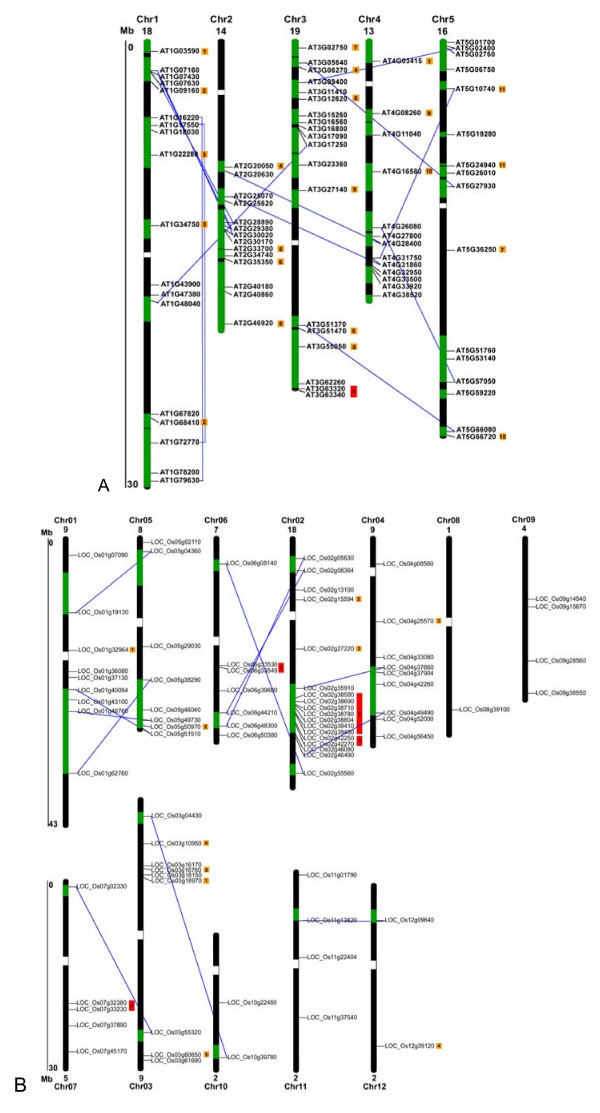
**Chromosomal distribution and segmental duplication events for PP2C genes in Arabidopsis (A) and rice (B)**. The chromosome number is indicated at the top of each chromosome representation. Green boxes indicate duplicated chromosomal segments retained from the most recent polyploidy event. Only duplicated regions containing PP2C genes are shown. Grey lines connect paralogous sister gene pairs in duplicated blocks. Potentially duplicated gene pairs as described in the text are marked with numbers in orange squares. Gene clusters that contain the members with high levels of sequence similarity, and likely have evolved via tandem duplications are marked with number in red rectangles.

To investigate the relationship between the genetic divergence within the PP2C family and gene duplication events in Arabidopsis, we located the 80 AtPP2C genes on the Arabidopsis chromosomes based on the location information provided in the TAIR database [60]. The genes are distributed across all five chromosomes except for the upper regions of chromosome 2 (Figure 4). Only one gene cluster was identified on chromosome 3, composed of AT3G63320 and AT3G63340, which share high level of sequence similarity and have thus likely resulted from a tandem gene duplication event. Thirteen paralogous AtPP2C pairs in the AtPP2C gene family were found on segmentally duplicated chromosomal regions (Figure 4A, blue lines), which was originated from a polyploidy event occurred around 24 to 40 million years ago, probably close to the emergence of the crucifer family [61-64]. The putative protein sequences of those 13 pairs were clustered with bootstrap value higher than 70% in the phylogenetic tree (Figure 1). An additional 11 paralogous gene pairs were identified based on phylogenetic relationship and structural similar (Figure 4A, orange squares). Although the chromosomal regions hosting these 11 gene pairs were not included by Blanc et al [61] as recently duplicated segmental regions, they were considered to be duplicated genes. Together, these genes represent 60% of all *AtPP2C*s, indicating that large-scale segmental duplication events have contributed largely to the current complexity of the PP2C gene family in Arabidopsis. Only 2.5% of *AtPP2Cs *(one pair) are organized in clusters, and likely have evolved via local or tandem duplications.

To determine the genomic distribution of the OsPP2C genes, the DNA sequence of each OsPP2C gene was used to search the rice genome database using BLASTN. Although all of the 12 rice chromosomes do contain OsPP2C genes, the distribution appears not random (Figure 4B). Relatively high densities of OsPP2C genes were found in specific chromosomal regions, including the top of chromosome 3, and the bottom of chromosome 2. In contrast, several large chromosomal regions are devoid of OsPP2C genes, such as the top parts of chromosomes 8, 9, and 10 and the central sections of chromosomes 7, 8, and 12. To detect possible relationships between OsPP2C genes and potential genome duplication events, we mapped 17 paralogous pairs of OsPP2C genes. Twelve of these pairs are linked to each of at least 8 previously predicted chromosomal/segmental duplications (Figure 4B, green bars, linked with blue lines) [54,65,66]. Five additional pairs of OsPP2C genes are close paralogs (Figure 4B, orange squares), but are located in the regions that have not been proposed to be the result of rice genome duplication events. Thus, 44% of the OsPP2C family might have evolved from putative rice genome duplication events. Four OsPP2C gene clusters contain the members with high levels of sequence similarity (Figure 4B, red rectangles), representing a total of 13 OsPP2C genes. Thus 17% of *OsPP2Cs *are organized in clusters and likely have evolved via local or tandem duplications.

Taken together, it appears that the PP2C gene families expanded differently in rice and Arabidopsis. Whole genome and chromosomal segment duplications mainly contributed to the expansion of both OsPP2Cs and AtPP2Cs giving rise to 44% and 60% of PP2C genes, respectively. But tandem or local duplication occurred less frequently in Arabidopsis (one pair, 2.5% of all PP2Cs) than rice (four clusters, 17%). It is noteworthy that the members of divergent PP2C subfamilies in both Arabidopsis and rice are located within the same chromosomal region, whereas the members of the same subfamily are distributed in different chromosomal regions, suggesting that PP2C genes were distributed widely in the genome of the common ancestor of monocots and eudicots. Furthermore, in both species the duplicated pairs of PP2C genes appear to have been preferentially retained compared with other genes, since the density of duplicated genes retained in recently duplicated chromosomal segments was estimated to be only 28% on average [61]. This highlights the indispensable roles of PP2Cs in both monocots and eudicots.

### Protein motifs of PP2Cs in Arabidopsis and rice

To identify the motifs shared among related proteins within the PP2C family, the MEME motif search tool was employed [76]. We identified 11 motifs and their sequences are shown in Table 3; the distribution of these motifs in PP2C proteins in Arabidopsis and rice is shown in Figure 1 and 2. Some motifs are widespread among PP2C proteins (e.g. motifs 1, 2, 3, 4, and 8). In contrast, other motifs are specific to only one or two subfamilies. For example, motifs 5 and 11 are specific to subfamilies C and D, respectively, while motif 9 is found almost exclusively in subfamily D members. Vice versa, motifs 6 and 7 are present in subfamily A, but were not found in subfamily D. Subfamilies C and D have especially distinctive characteristics in terms of motif distribution within the PP2C family.

**Table 3:** Sequences and lengths of conserved motifs in the amino acid sequences of Arabidopsis PP2C genes

Motif	Width	Multilevel consensus sequence
1	21	DEFLILASDGLWDVMSNQEAV
2	21	WRVKGQLAMSRAFGDWYLKQY
3	15	LYVANVGDSRAVLCR
4	15	HFFGVYDGHGGPHAA
5	57	DIVHNHPRNGIAQRLIKAALQEAAKKREMRYHDLKKIPQGVRRHYHDDITVIVIFLD
6	21	DHKPNRPDERERIEKCGGYVF
7	21	LTEEALQRGSKDNITCIVVCF
8	11	GSTCVTAIICG
9	41	GRQDGLLWYKDLGQHAAGEFSMAVVQANNLLEDQCQVESGP
10	41	YCKKHLFENILKHPDFWTDTKNAIKNAYKQTDQYFLESCSW
11	29	AEQLSTEHNTNIEEVRQELKSLHPDDPQI

The different motif distribution in the respective protein sequences may provide an anchor to study the divergence of gene function in different subfamilies. Furthermore, the fact that proteins in the same subfamily exhibit highly similar motif distribution supports their close evolutionary relationship.

### Expression patterns of PP2C genes in Arabidopsis and rice

**Figure 5:**
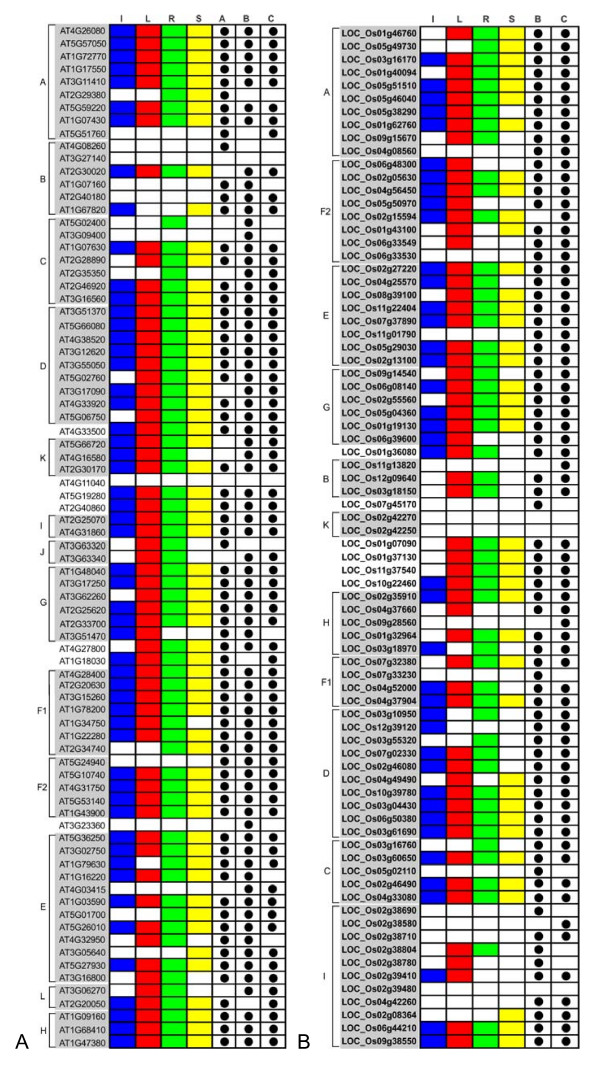
**The expression patterns in different tissues for AtPP2C and OsPP2C genes**. Capital letters on the left indicates the major subfamilies. PP2Cs are aligned in the same order as in the corresponding phylogenetic trees. Subfamilies of PP2C genes are highlighted in grey. The expression data of AtPP2C genes in different tissues were combined from microarray (derived from Genevestigator), MPSS, and EST abundance data. The expression data of OsPP2C genes were extracted from MPSS and EST abundance data only. Letters on the top indicate different tissues and databases. A, B and C represent microarray, MPSS and EST data, respectively. A positive signal is indicated by a colored box for the following tissues: blue for inflorescences (I), red for rosette leaves (L), green for roots (R), and yellow for siliques (S). The white boxes indicate that no expression could be detected.

Gene expression patterns can provide important clues for gene function. To date, a comparative analysis of the expression patterns of the whole PP2C gene family has not been performed. Therefore, we firstly examined the expression of AtPP2C genes in inflorescence, leaf, root and silique tissues using microarray data from Genevestigator [67], massively parallel signature sequencing (MPSS) data [68] and EST abundance data from NCBI [69] (Figure 5A). Expression profiles for 67, 73, and 68 PP2C genes were extracted from the Genevestigator, MPSS, and EST databases, respectively. After integrating the three data sets, we found that most of the genes have a very broad expression spectrum, and only ten genes (AT1G07160, AT2G40180, AT3G09400, AT3G27140, AT3G23360, AT4G03415, AT4G08260, AT4G11040, AT5G24940, and AT5G51760) were not detected in any of those four tissues. However, transcript evidence for all genes is present in the mix tissue samples from Arabidopsis with the sole exception of AT4G11040 and AT3G27140, indicating that they might be expressed only at highly specific developmental stages. Since no evidence for the expression of AT3G27140 was found in full-length cDNA datasets at NCBI either (Table 1), it might be a pseudogene. We also summarized the expression of rice PP2C genes in leaf, flower, root, seed and stem tissues using the same approach (Figure 5B). Expression signatures for 71 OsPP2C genes were detected in the MPSS database, and 69 members had matching ESTs. No expression evidence was found in any database for three OsPP2C genes (Os02g39480, Os02g42250, Os02g42270) (Table 2), suggesting that they might be pseudogenes. Twenty OsPP2C genes had no detectable tissue specific expression, but had matching transcript sequences (Figure 5B), which might thus be expressed at specific developmental stages or under special conditions only.

A total of 49 AtPP2C genes were expressed in all tissues examined. Only three AtPP2C genes (A2G35350, AT3G05640 and AT5G02440) displayed highly preferential expression in certain tissue (Figure 5). Twenty-seven OsPP2C genes were expressed in all tissues examined, and six OsPP2C genes showed tissue-specific expression. Most AtPP2C genes (84%) and OsPP2C genes (72%) were expressed in more than 2 tissues examined, suggesting that most PP2Cs in plants play functional roles at multiple developmental processes, and only a few members act in tissue-specific biological processes. It is interesting to note that 19 of 29 paralogous gene pairs (18 duplicated gene pairs in Arabidopsis and 11 in rice) show differential expression patterns, suggesting functional diversification of most duplicated gene pairs. This result is consistent with the previous findings that the expression of paralogs frequently diverged to become part of parallel networks, and that the expression of each gene is strongly correlated with other non-homologous genes acting in the corresponding networks, suggesting that neo-functionalization is a major feature of the long-term evolution of polyploids [61].

**Figure 6:**
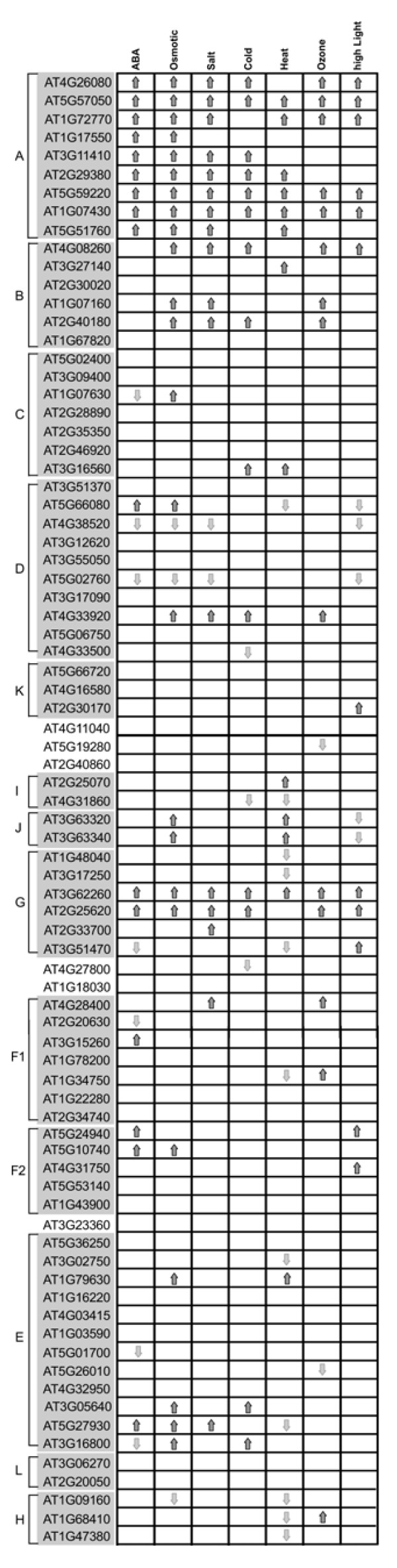
**Expression patterns in response to ABA treatment and diverse environmental stress stimuli of AtPP2C genes**. Microarray data for the AtPP2C genes in response to multiple environment stimuli or ABA treatment were extracted from microarray data provided in Genevestigator. Up- and down-regulated expression patterns of AtPP2C genes under ABA signal and six environmental stimuli (as indicated on top) are shown as upwards black and downwards grey arrows, respectively.

**Figure 7:**
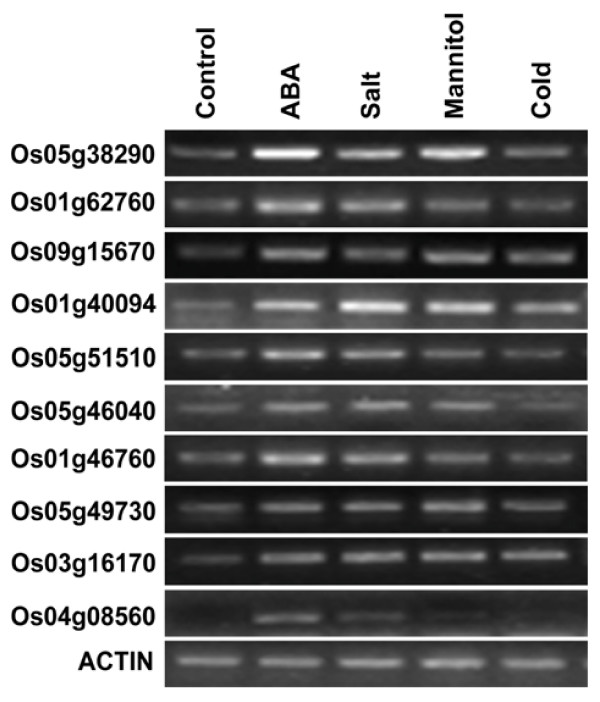
**Expression pattern analysis of subfamily A genes in rice in response to abiotic stress or ABA treatments**. Plants were grown on Murashige and Skoog (MS) agar medium for 10 days and were subsequently treated with ABA (100 μM), NaCl (150 mM), mannitol (150 mM), cold (4°C), or water (as a control). Total RNA was used for semi-quantitative RT-PCR using gene specific primers as indicated. The rice actin gene was used as an internal control.

The phytohormone abscisic acid (ABA) plays important regulatory roles in many stress and developmental responses throughout the plant life cycle, particularly in the ability to sense and respond to various unfavorable environmental conditions, including drought, salinity, cold shock, and wounding during vegetative growth [21,33,70-74]. To investigate possible involvement of PP2Cs in ABA mediated signaling, we examined the expression of all AtPP2Cs in response to ABA treatment and several environmental stimuli using the Genevestigator microarray dataset [67] (Figure 6). The expression of 16 PP2Cs was found to be up-regulated in response to ABA treatment, including all 9 members of subfamily A. Five AtPP2C genes belonging to subfamily A have been characterized as negative regulators in ABA-mediated processes, including AT4G26080 (*ABI1*), AT5G57050 (*ABI2*), AT1G72770 (*HAB1*), AT1G17550 (*HAB2*), and AT3G11410 (*ATPP2CA*) [16-20]. Unlike the PP2C genes from other subfamilies, all members from subfamily A were induced by several stimuli (Figure 6). To explore whether also OsPP2C genes from subfamily A play functional roles in ABA mediated signaling pathways and stress responses, we analyzed the expression of 10 OsPP2C genes in rice plants treated with ABA, salt, osmotic (mannitol) and cold stress by reverse transcriptase polymerase chain reaction (RT-PCR). The results of our experiment indicate elevated expression levels of all OsPP2C genes from subfamily A upon treatment with ABA and salt (Figure 7). Also mannitol and cold treatment led to induced expression of several subfamily A members, but expression and induction levels differed among these genes (Figure 7). Thus, the expression pattern of the OsPP2C subfamily A genes is in good agreement with the microarray data for Arabidopsis subfamily A members, suggesting that the members of this subfamily play foremost roles in ABA-mediated processes related to stress responses both in monocots and eudicots.

Interestingly, the expression of 7 PP2C genes in Arabidopsis was suppressed by ABA treatment, and 2 of them are the members of subfamily D (Fig. 6). In previous studies, a PP2C from *Fagus sylvatica *(FsPP2C2) was described as a positive regulator of the ABA signal transduction pathway, contrary to most other PP2Cs described so far [34]. Based on phylogenetic reconstructions, FsPP2C2 belongs to subfamily D and is most closely related to AT3G51370 (data no shown). This result suggests that at least some members of subfamily D might have a positive role in ABA signaling pathways.

The expression of 30 AtPP2C genes was not influenced by different environmental stimuli (Fig. 6), suggesting that 38% of AtPP2Cs might not play functional roles in response to environmental stress signals. Among them, five genes are from subfamily C, which is composed of *POLTERGEIST *(*POL*, AT2G46920), five *POL*-like genes (*PLL1-PLL5*, gene IDs in Table 1) and AT3G16560. *POL *and *PLL1 *have been shown to regulate meristem development and pedicel length, while *PLL4 *and *PLL5 *were found regulating leaf development [39,75]. These results indicate that at least some of these 38% AtPP2Cs (e.g. some members of subfamily C) might be involved in developmental processes.

In summary, our expression analyses provide evidences that PP2C genes in different subfamilies might play different functional roles in distinct signaling pathways.

#### Upstream regulatory regions of subfamily A PP2C genes in Arabidopsis and rice

Promoters in the upstream region of genes play key roles in conferring developmental and/or environmental regulation of gene expression. To further elucidate the transcriptional regulation machineries of the 19 PP2C genes in subfamily A in Arabidopsis and rice, two approaches were attempted in this study.

First, we analyzed the 1000-base-pair (bp) upstream promoter sequences of these AtPP2C genes using the PlantCARE database [77], and validated the results using the PLACE databases [78]. Based upon this approach, a total of 20 types of *cis*-regulatory elements (CREs) were identified among the 19 subfamily A AtPP2Cs, which do not contain the common eukaryotic regulatory elements, such as TATA and CAAT boxes. The names and functions of these 20 motifs are shown in Table 4. Their amounts and positions in every subfamily A member obtained by the first method are displayed in Additional Files 1 and 2.

Second, we used these 20 motifs as a base for constructing their frequency matrix following a frequency distribution approach (see Methods and [79]), to compare the frequency of these motifs in the promoter regions of subfamily A with that in the whole genome, in order to explore whether these 20 motifs are overrepresented in subfamily A in Arabidopsis and rice. The frequency matrices of 9 motifs (ABRE, CE3, G-box, GCN4 motif, HSE, O_2_-site, TC-rich repeats, TCA-element and TGACG motif) were generated. Only a very limited number of sequences containing the remaining 11 motifs (ACE, ARE, AuxRR-core, CAT-box, CCGTCC-box, CGTCA motif, circadian, LTR, motif 1, Skn-1 motif, 5UTR Py-rich stretch) are present in the PLACE, Plantcare and other databases, therefore their frequency matrices could not be generated in this analysis. Thus, only the frequency matrices of 9 motifs were used to identify all single occurrences of these motifs in the promoter regions of the subfamily A members and all protein-coding genes using the genomic sequences of Arabidopsis (genome release 7.0, January 2007) [60] and rice (release 4.0, January 2006) [47] (Table 5). Our results suggest that in both species, 5 out of 9 motifs (GCN4, HSE, O_2_-site, TC-rich repeats and TCA element) are not over-represented in the upstream regions of PP2C subfamily A members. In contrast, in both species the frequency of ABREs (involved in ABA responsiveness) is much higher in the promoter regions of subfamily A than in the whole genome (Tab. 5). The CE3 (involved in ABA and VP1 responsiveness) and G-box (involved in light responsiveness) motifs were found only in subfamily A promoters from rice but absent in those from Arabidopsis. Our results support a previous finding that CE3s are abundant in rice but almost absent in Arabidopsis [79]. In reverse, the TGACG motif (involved in MeJA responsiveness) appeared relatively abundant in subfamily A promoters from Arabidopsis while absent in those from rice. The different motif distributions in Arabidopsis and rice point to evolutionary divergent patterns of PP2C gene regulatory networks. The abundance of ABA responsive elements in subfamily A PP2C gene promoters provides additional evidences for their role in ABA signaling, and reveals shared (ABRE) as well as different (CE3) gene regulation machineries for ABA responsiveness in dicots and monocots. The occurrence of other motifs like G-box suggests that some subfamily A PP2C genes might be involved also in other biological processes, such as light-responsive pathways.

**Figure 8:**
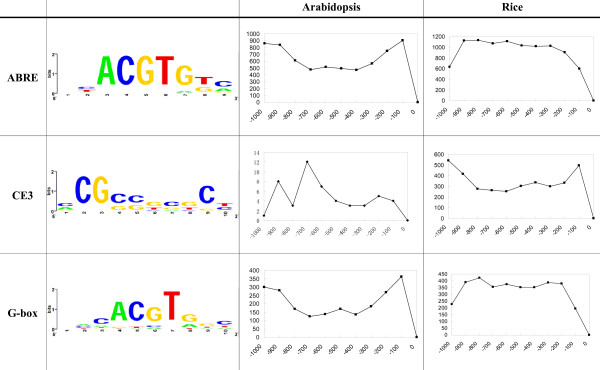
**Sequence logos and distribution of ABREs, CE3s and G-boxes on the upstream of peptide-coding genes in Arabidopsis and rice**. The first column shows the names of each cis-regulatory element (CRE). The second column for each motif shows the nucleotide frequency distribution graphed using WebLogo, where the sizes of the characters represent the frequencies of occurrence. The third column provides a graphic representation of the frequency distribution (y-axis) of each motif in the [-1000, -1] regions (x-axis).

Additional information on the frequency distributions of ABRE, CE3 and G-box in the 1000 bp upstream regions and their nucleotide frequency distributions in Arabidopsis and rice are provided in Fig. 8. ABRE, CE3 and G-box are widely distributed in the upstream regions of subfamily A as well as in the whole genome of Arabidopsis and rice (data no shown).

**Table 4:** Putative regulatory *cis*-element sequences in 5'-upstream regions of subfamily A members

Symbol	Name	Description
A	ABRE	cis-acting element involved in the abscisic acid responsiveness
B	ACE	cis-acting element involved in light responsiveness
C	ARE	cis-acting regulatory element essential for the anaerobic induction
D	AuxRR-core	cis-acting regulatory element involved in auxin responsiveness
E	CAT-box	cis-acting regulatory element related to meristem expression
F	CCGTCC-box	cis-acting regulatory element related to meristem specific activation
G	CGTCA-motif	cis-acting regulatory element involved in the MeJA-responsiveness
H	CE3	cis-acting element involved in ABA and VP1 responsiveness
I	circadian	cis-acting regulatory element involved in circadian control
J	G-box	cis-acting regulatory element involved in light responsiveness
K	GCN4_motif	cis-regulatory element involved in endosperm expression
L	HSE	cis-acting element involved in heat stress responsiveness
M	LTR	cis-acting element involved in low-temperature responsiveness
N	motif I	cis-acting regulatory element root specific
O	O_2_-site	cis-acting regulatory element involved in zein metabolism regulation
P	Skn-1_motif	cis-acting regulatory element required for endosperm expression
Q	TC-rich repeats	cis-acting element involved in defense and stress responsiveness
R	TCA-element	cis-acting element involved in salicylic acid responsiveness
S	TGACG-motif	cis-acting regulatory element involved in the MeJA-responsiveness
T	5UTR Py-rich stretch	cis-acting element conferring high transcription levels

**Table 5:** Genome-wide and PP2C subfamily A occurrence of *cis*-regulatory elements (CREs) in the upstream regions of Arabidopsis and rice.

CRE	Species	Genomic data set				Subfamily A data set			
		
		Real		Random		Real		Random	
		
		Genomic data set	Frequency	Randomized d data set	Frequency	Subfamily A data set	Frequency	Randomized data set	Frequency
ABRE	A. thaliana	6492	24.21%	3449	12.86%	12	133%	1	11.1%
	O. sativa	9662	23.52%	7331	17.85%	10	100%	1.50	15%
G-box	A. thaliana	2129	7.94%	211	0.79%	0	0	0	0
	O. sativa	3440	8.37%	1109	2.70%	5	50.00%	0.60	6.0%
CE3	A. thaliana	40	0.15%	32	0.12%	0	0	0	0
	O. sativa	3527	8.59%	634	1.54%	1	10.00%	0.33	3.3%
GCN4	A. thaliana	1029	3.84%	961	3.58%	0	0	0.5	5.56%
	O. sativa	950	2.31%	1354	3.30%	0	0	0	0
HSE	A. thaliana	32	0.12%	15	0.06%	0	0	0.1	1.11%
	O. sativa	42	0.10%	7	0.02%	0	0	0	0
O2	A. thaliana	666	2.48%	320	1.19%	0	0	0	0
	O. sativa	1509	3.67%	538	1.31%	0	0	0.30	3.0%
TC-rich	A. thaliana	4691	17.49%	2652	9.89%	0	0	0.8	8.89%
	O. sativa	3765	9.17%	1996	4.86%	0	0	0	0
TCA	A. thaliana	1581	5.90%	255	0.95%	0	0	0	0
	O. sativa	2614	6.36%	487	1.19%	0	0	0	0
TGACG	A. thaliana	4139	15.43%	4005	14.93%	5	55.56%	1.20	13.33%
	O. sativa	9221	22.45%	10104	24.60%	0	0	1.70	17.0%

Our results also indicate that although the mapping of known motifs to the promoter regions of genes (i.e. our first approach) can reveal interesting patterns and insights, it is necessary to compare the frequency of these motifs in the genes against their genome-wide frequency (i.e. our second approach), in order to explore whether these motifs are truly enriched in the promoter regions of these genes and to eliminate false positive motif instances.

## Conclusion

We provide here a comparative genome-wide overview of the PP2C family of Arabidopsis and rice, one of the largest gene families in plants. Our computational studies show that despite the big differences in genome sizes of Arabidopsis and rice, the two species appear to have very similar numbers of genes encoding PP2C proteins (80 and 78, respectively), which are much higher than in yeast and human. The AtPP2Cs and Os PP2cs are divided into 13 and 11 subfamilies, respectively, each of which shares a monophyletic origin, common protein motifs and intron/exon structures. We identified both common and lineage-specific subfamilies and potential 'gene birth-and-death' events in the PP2cs of these two species. Our analyses suggest that whole genome and chromosomal segment duplications mainly contributed to the expansion of both OsPP2Cs and AtPP2Cs, but tandem or local duplication occurred less frequently in Arabidopsis than rice. Our protein motif analysis detected the motifs widespread among the PP2C proteins as well as the motifs specific to only one or two subfamilies. Our computational and experimental expression analyses suggest that 1) most PP2C genes play functional roles in multiple tissues in both species, 2) most genes in subfamily A play primary roles in stress tolerance, especially ABA response, 3) subfamily D members may constitute positive regulators in ABA-mediated signaling pathways. We further employed two approaches to analyze the putative upstream regulatory elements of PP2C subfamily members. The results obtained by both methods support the function of subfamily A in ABA responses, and provide insights into the shared and different transcriptional regulation machineries as well as evolutionary divergent patterns of PP2C gene regulatory networks in dicots and monocots. Overall, our study provides comprehensive information and novel insights into the functions and regulatory mechanisms, as well as the evolution and divergence of the PP2C genes in dicots and monocots. The results presented here lay a solid foundation for future studies on the functional divergence of different PP2c subfamilies.

## Materials and methods

### Identification of PP2C genes in Arabidopsis and rice

To collect all members of the PP2C gene family in Arabidopsis, an initial keyword search was performed using "protein phosphatase 2C" to screen six databases, i.e. NCBI [69], DATF [80], MAtDB [81], TAIR [60], TIGR [47] and PlantsP [82]. The resulting protein hits were pooled together and redundant sequences were removed using a custom Perl program. Remaining proteins sequences were then used as a query to perform multiple database searches against proteome and genome files downloaded from TAIR. Stand-alone versions of BLASTP and TBLASTN available from NCBI were used with the e-value cutoff set to 1e-003. After eliminating redundant sequences, the obtained protein sequences were screened for the existence of a PP2C catalytic domain by the hidden Markov model provided with the SMART [45] and Pfam [46] tools. Proteins without a PP2C catalytic domain were eliminated from the dataset.

To collect all members of the PP2C gene family in rice, all predicted AtPP2C protein sequences were initially used as query sequences to search against the TIGR rice database [47] using BLASTP. Multiple BLAST searches using protein sequences of significant hits as the queries were then performed in several rice databases, including NCBI [69], TIGR [47], Rice Genome Database-japonica of the Rice Genome Research Program [83] and the International Rice Genome Sequencing Project (IRSGP) [84]. After removal of redundant sequences from our data set, SMART and Pfam were used to confirm the presence of PP2C catalytic domains as described above.

### Alignment and phylogenetic analysis of PP2C sequences

Multiple alignments of amino acid sequences were generated using ClustalX (1.83) and were manually corrected. Phylogenetic trees were constructed by neighbor-joining algorithms of Mega (4.0). Bootstrapping was performed 1000 times to obtain support values for each branch.

### Chromosomal localization and duplication of PP2C genes

To determine the location of PP2C genes on the five Arabidopsis chromosomes, the Chromosome Map Tool from TAIR was used [85]. Gene duplications and their presence on duplicated chromosomal segments were investigated using "Paralogous in Arabidopsis" with the default parameters set to a minimum threshold for paired proteins per block above 7 [86]. For rice, all the sequenced contigs of *japonica *cultivar Nipponbare has been physically reconstructed as pseudomolecules by the IRGSP representing the 12 rice chromosomes, which are available in GeneBank. Each of the rice PP2C genes was positioned on these rice chromosome pseudomolecules using BLASTN. For the detection of large segmental duplications, we refer to the duplicated blocks map generated by Xiong et al. [66].

### Motifs identification of PP2C proteins in Arabidopsis and rice

To identify motifs shared among related proteins within PP2C gene family, we used the MEME motif search tool [76] with default settings except that the maximum number of motifs to find was defined as 20 and the maximum width was set to 300.

### *In silico *expression analysis of PP2C genes

cDNA signatures from MPSS [68] and EST abundance data from NCBI [87] were used to detect the expression patterns of Arabidopsis and rice PP2C genes in different organs. Microarray data from Genevestigiator [67] were used to determine the expression profile of Arabidopsis PP2C genes in different organs and under multiple environmental stimuli.

### RT-PCR analysis of PP2C gene expression

Plant tissues of the rice *japonica *cultivar Nipponbare were ground in liquid nitrogen. For reverse transcriptase (RT) mediated PCR analysis, total RNA was isolated using the Trizol (Invitrogen, Carlsbad, CA, USA) according to the manufacturer's instructions. RNA preparations were then treated with DNase I (Promega, Madison, WI, USA). First strand cDNA synthesis was performed by using oligo (dT) primer and M-MLV RT (Promega, Madison, WI, USA). PCR products were fractionated on 1% agarose gels containing ethidium bromide and photographed under UV light. These experiments were independently replicated at least three times under identical conditions. Details of primers are listed in the additional file 3.

### Upstream sequences

A total of 32,041 1000-bp upstream sequences from Arabidopsis were downloaded from TAIR 7.0 [60]. All upstream sequences that correspond to genes that do not code for proteins or oligopeptides were discarded (5012 sequences). Then, the reverse complementary sequences were obtained. The final database of Arabidopsis 1-kb upstream sequences consisted of 54,058 individual sequences. A database of 66,170 1000-bp upstream sequences from rice was downloaded from TIGR 4.0 [47]. Upstream sequences were cleared of transposable elements and related gene models using the gene identifiers of transposable-element-related gene models provided by TIGR (15,424 gene identifiers). After obtaining reverse complementary sequences, the file used for the screening consisted of 82,144 sequences. The 1000-bp upstream sequences of PP2C genes in subfamily A were extracted from the final databases from Arabidopsis and rice.

### Randomized sequences

Randomized sequences were generated by shuffling the extracted upstream sequences using the EMBOSS program "shuffleseq" [88]. During the shuffling procedure, the single-nucleotide frequency of each sequence is maintained, but the nucleotide order is randomly shuffled. The reshuffling process was repeated 10 times to generate 10 different data sets of randomized 1-kb upstream sequences for Arabidopsis and rice, respectively.

### Searching single occurrences of CREs

The subfamily A, genomic and randomized data sets were screened for single occurrences of CREs on the plus and minus strands, using the frequency matrices and the program "Profit" from EMBOSS [88]. For each screened data set the program generates a list of matches, including the sequence identifier, element, score and position for every match. The score is calculated from the frequency matrix. An arbitrary 95% cut-off was used. The position of a match is expressed in terms of the distance to the translation initiation codon (ATG).

## Authors' contributions

TX and DW carried out all the data analyses. TX performed the RT-PCR experiments. FN and YZ contributed the PP2C gene family background knowledge. YZ and CZ provided financial support for the study. TX and SZ jointly wrote the draft manuscript. SJ, SZ and FN double checked the presented data. XT, YZ, JE and CZ participated in the writing of the final version of the manuscript.

## Supplementary Material

Additional file 1 **Table S1 Distribution of putative regulatory *cis*-elements in 5'-upstream regions of subfamily A members.** Click here for file

Additional file 2 **Figure S1 Putative *cis*-elements distribution in upstream regulatory regions in PP2C subfamily A and D members from Arabidopsis and rice**. 1000 bp genomic DNA sequences upstream of the first exon of each gene were extracted and the positions of the upstream regulatory regions are indicated. The relative positions of *cis*-elements are labeled with capital letters, which are annotated at the bottom. Click here for file

Additional file 3 **Table S2 Oligonucleotide primers for subfamily A members in rice used for RT-PCRs.** Click here for file
