# Supplementary material for: Genome-wide and expression analysis of protein phosphatase 2C in rice and Arabidopsis
Source: BMC Genomics. 2008 Nov 20;9:550. doi: 10.1186/1471-2164-9-550 (PMC2612031; doi:10.1186/1471-2164-9-550)
Supplement: Additional file 1 — Table S1 Distribution of putative regulatory cis-elements in 5'-upstream regions of subfamily A members. [file 1471-2164-9-550-S1.doc]

**Table S1. Distribution of putative regulatory *cis*-elements in 5’-upstream regions of subfamily A members**

| Gene identifer | ABRE | ACE | ARE | AuxRR-core | CAT-box | CCGTCC-box | CGTCA-motif | CE3 | circadian | G-box | GCN4_motif | HSE | LTR | motif I | O2-site | Skn-1_motif | TC-rich repeats | TCA-element | 5UT Py-rich stretch | TGACG-motif |
| --- | --- | --- | --- | --- | --- | --- | --- | --- | --- | --- | --- | --- | --- | --- | --- | --- | --- | --- | --- | --- |
| AT4G26080 | 1 |  | 4 |  |  |  | 2 |  |  | 2 | 1 |  |  |  |  | 6 | 1 | 1 | 1 | 1 |
| AT5G57050 | 3 | 1 |  |  |  |  |  |  | 1 | 2 |  |  |  |  |  | 2 | 2 |  |  |  |
| AT1G72770 | 3 | 1 | 3 |  |  |  | 2 |  | 1 | 6 |  | 1 |  |  |  |  | 1 | 1 |  | 1 |
| AT1G17550 | 1 |  | 2 |  |  |  | 2 | 1 |  | 3 | 2 |  |  |  | 1 | 3 |  |  |  | 2 |
| AT3G11410 | 4 |  | 1 |  |  |  | 2 |  | 1 | 4 |  | 1 |  | 1 |  |  | 2 | 1 |  | 2 |
| AT2G29380 | 1 | 2 | 1 |  |  |  | 2 |  | 1 | 5 |  |  |  |  | 1 | 4 | 2 | 1 | 1 | 2 |
| AT5G59220 | 3 | 2 |  |  | 4 |  |  |  |  | 3 |  |  |  |  |  | 5 |  |  |  | 4 |
| AT1G07430 | 6 | 5 | 1 | 1 |  |  |  |  |  | 10 |  | 1 | 1 |  |  | 1 |  |  |  |  |
| AT5G51760 | 3 | 3 | 3 |  |  |  | 3 |  | 1 | 7 |  | 1 | 2 |  |  | 2 | 2 |  |  | 2 |
| Os01g46760 | 1 | 3 |  |  |  |  |  |  | 1 | 5 |  |  |  |  |  | 1 | 1 |  |  |  |
| Os05g49730 | 7 | 3 | 2 | 1 | 1 |  | 1 |  |  | 8 |  |  | 1 |  |  |  |  |  |  | 1 |
| Os03g16170 | 8 | 2 | 1 |  |  |  | 2 | 1 |  | 13 |  | 1 | 1 | 1 | 1 |  |  | 1 |  | 1 |
| Os01g40094 | 5 | 1 | 1 |  |  | 1 | 1 | 1 | 1 | 13 |  |  | 1 |  | 1 |  | 1 |  |  | 1 |
| Os05g51510 | 4 |  |  |  |  |  | 1 |  | 1 | 2 |  | 1 |  |  |  |  |  |  |  | 1 |
| Os05g46040 | 1 | 1 | 1 |  | 1 |  |  |  |  |  |  | 1 |  |  |  |  | 1 |  |  |  |
| Os05g38290 | 11 | 1 | 1 |  |  | 1 | 1 |  |  | 20 |  |  |  |  | 1 | 1 |  |  | 3 | 1 |
| Os01g62760 | 4 | 1 |  |  |  | 1 | 1 | 1 |  | 13 |  |  |  |  |  |  |  |  |  | 1 |
| Os09g15670 | 8 | 1 | 1 |  | 1 |  |  | 1 | 1 | 6 |  | 1 |  |  |  | 1 | 1 | 1 |  |  |
| Os04g08560 | 3 | 2 |  |  |  |  | 1 | 1 |  | 3 |  |  |  |  |  | 1 | 1 |  |  | 1 |
