# Supplementary figures and images for: Genome-wide and expression analysis of protein phosphatase 2C in rice and Arabidopsis
Source: BMC Genomics. 2008 Nov 20;9:550. doi: 10.1186/1471-2164-9-550 (PMC2612031; doi:10.1186/1471-2164-9-550)

**
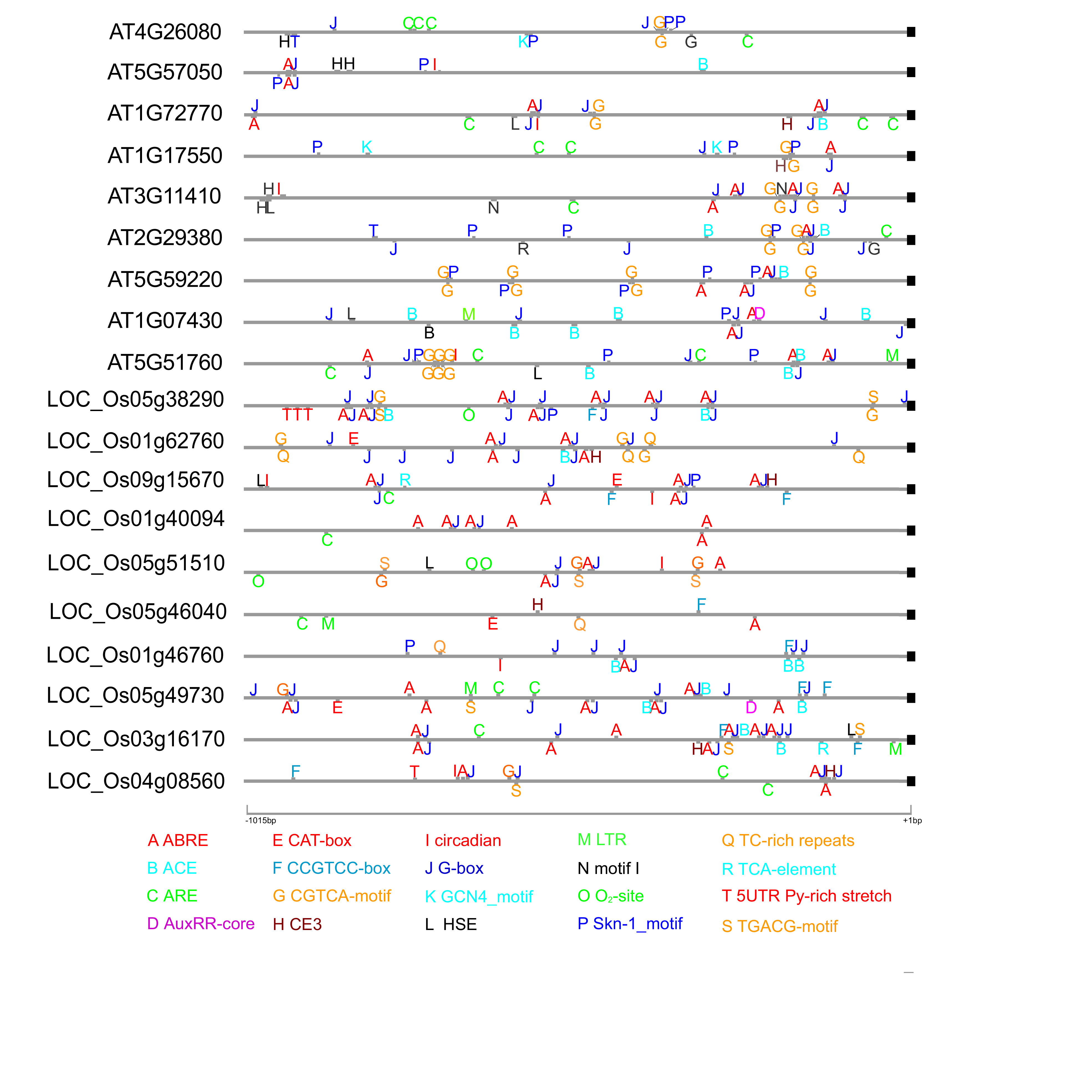
**

**Figure S1**

Supplement: Additional file 2 — Figure S1 Putative cis-elements distribution in upstream regulatory regions in PP2C subfamily A and D members from Arabidopsis and rice. 1000 bp genomic DNA sequences upstream of the first exon of each gene were extracted and the positions of the upstream regulatory regions are indicated. The relative positions of cis-elements are labeled with capital letters, which are annotated at the bottom. [file 1471-2164-9-550-S2.doc]
