# Supplementary material for: Genome-wide and expression analysis of protein phosphatase 2C in rice and Arabidopsis
Source: BMC Genomics. 2008 Nov 20;9:550. doi: 10.1186/1471-2164-9-550 (PMC2612031; doi:10.1186/1471-2164-9-550)
Supplement: Additional file 3 — Table S2 Oligonucleotide primers for subfamily A members in rice used for RT-PCRs. [file 1471-2164-9-550-S3.doc]

**Table S 2 Primers of subfamily A in rice for RT-PCRs**

| gene ID | 5'-Primer | 3'-Primer |
| --- | --- | --- |
| LOC_Os01g40094 | AACAGGCGTTTGTGGACTGCTT | TTGCGAGCAACATCGCATACCT |
| LOC_Os03g16170 | TGAAGCCGTACGTGATATGCGA | ACGCTGACATTGTCCGAGGTTT |
| LOC_Os05g49730 | AGGACTTCTTCGCGGTGTACGA | TTCCAGTTGATGACTCTGCCTCCA |
| LOC_Os01g46760 | ATCCTCGGTGTCCTTGCTACTT | TTCTCTTTGCTCGCCGTCGTTCTA |
| LOC_Os05g51510 | AGTCCAAGAAGCTGTGGGAGAA | TTTGCACGAGCGACAACTGTGA |
| LOC_Os05g46040 | TGCGCTTGTCTGCTCATCTCAT | TGCGACTTTGCATGCCTCTTCA |
| LOC_Os05g38290 | AAGGACGTCATGGAGAAAGGGT | AGGATCAGGCACTCATCGTCGT |
| LOC_Os01g62760 | AGGACAGGATGCACGAGATTGT | TCGGGTTTGTGGTCAACGGATA |
| LOC_Os09g15670 | TGCAGCTATCCTCCGACCACAA | TCGACGACGACGACAGAGATGTT |
| LOC_Os04g08560 | 5AAGGGCCTTAATCCATCGCTTTCC | TGAGATCACATCCCACATCCCA |
